# The Effect of Charcoal-Based Dentifrice and Conventional Whitening Toothpaste on the Color Stability and Surface Roughness of Composite Resin: A Systematic Review of In Vitro Studies

**DOI:** 10.3390/dj12030058

**Published:** 2024-03-01

**Authors:** Cody A. Wiktorski, Dimitrios Michelogiannakis, P. Emile Rossouw, Fawad Javed

**Affiliations:** Department of Orthodontics and Dentofacial Orthopedics, Eastman Institute for Oral Health, University of Rochester, Rochester, NY 14620, USA; cody_wiktorski@urmc.rochester.edu (C.A.W.); dimitrios_michelogiannakis@urmc.rochester.edu (D.M.); emile_rossouw@urmc.rochester.edu (P.E.R.)

**Keywords:** charcoal-based dentifrices, composite resin, composite-based restorations, color stability, surface roughness, whitening toothpaste

## Abstract

The objective was to systematically review studies that evaluated the effect of charcoal-based dentifrices (CbDs) and conventional whitening toothpastes (CWTs) on the color stability (CS) and/or surface roughness (SR) of composite resin (CR). The question we focused on was “Do CbD and CWT affect the CS and/or SR of CR?” Indexed databases were searched without language and time restrictions up to and including May 2023 using different keywords. Original experimental studies were included. The risk of bias (RoB) was assessed using the Quality Assessment Tool for In Vitro Studies. Ten in vitro studies performed on CR were included. The number of CR samples assessed ranged between 18 and 200. In one study, CbDs altered the CS and SR of CR, whereas another study showed no difference in changes in the SR and CS of CR when CbDs were compared with CWTs. One study showed that compared with CWTs, CbDs caused changes in the CS of CR but changes in SR were similar between the two dentifrices. One study showed that CbDs and CWTs improved the overall color and enhanced the SR of CR. Three studies had a high RoB, five had a medium RoB, and two had a low RoB. Compared to CWTs, CbDs appeared to affect the CS of CR, but the SR of CR induced by both dentifrices remained consistent. Further well-designed and power-adjusted studies are needed.

## 1. Introduction

Composite resin stands as a cornerstone in modern restorative dentistry, offering a balance between aesthetics, functionality, and versatility [1,2]. Its widespread use underscores its significance in providing patients with durable and aesthetically pleasing dental restorations. The resin matrix is typically composed of a bisphenol-A glycidyl methacrylate or urethane dimethacrylate monomer. These monomers undergo polymerization, creating a durable and stable resin matrix. Inorganic filler particles, such as silica or glass, are added to reinforce composite resin and enhance its mechanical properties. Within the domain of dental restorations, CR assumes a central role by seamlessly conforming to the shape and bonding requirements of fractured or decayed teeth, thereby achieving a harmonious integration of function and esthetics. However, the success of composite resin (CR) restorations extends beyond immediate aesthetic considerations, encompassing nuanced factors such as color stability (CS) and the enduring maintenance of a smooth surface texture over time [3,4,5,6,7,8,9,10,11,12]. CS, a critical facet in the longevity of dental restorations, pertains to a material’s resistance to color alterations induced by diverse factors, including environmental exposure, dietary habits, and oral hygiene practices [13]. In other words, the CS of CR holds significant clinical implications. The long-term CS of CR ensures that restorations maintain a natural appearance, avoiding the need for premature replacements. Patients benefit not only from the functional success of the restoration but also from sustained aesthetic satisfaction. Concurrently, surface roughness (SR) emerges as a decisive determinant influencing plaque retention, wear, and overall aesthetics, thereby shaping the comprehensive success of CR from both a functional and aesthetic standpoint [11]. Daily oral hygiene practices, including the selection of dentifrices, constitute a network of factors significantly impacting the durability and clinical performance of composite-based restorations [11,14,15]. In summary, the SR of CR influences its wear resistance and overall durability. A smoother surface exhibits reduced wear and abrasion, contributing to the longevity of the restoration. In high-stress areas such as occlusal surfaces, a polished and wear-resistant CR surface is essential to withstand the mechanical forces experienced during mastication, ensuring the restoration’s structural integrity over time.

The recent upsurge in popularity of charcoal-based dentifrices (CbDs) introduces an intriguing dimension to the landscape of dental care, particularly among individuals actively seeking more affordable and easily accessible teeth-whitening solutions [16,17]. Activated charcoal, a pivotal constituent of CbDs, is acclaimed for its purported ability to adsorb extrinsic stains, plaque, and debris, offering the promise of a brighter and whiter smile [18]. Manufacturers posit that CbDs’ natural and effective stain removal properties emanate from the porous nature of activated charcoal [17,19]. However, as the utilization of CbDs continues to escalate, legitimate concerns have surfaced regarding their potential impact on the physical and visual characteristics of CR. The nuanced tapestry of experimental investigations [3,4,5,6,7,8,9,11,12] seeking to evaluate the influence of CbDs on the SR and CS of CR has woven a complex narrative, with conflicting interpretations. Torso et al. [8] suggested that chronic exposure of CR to CbDs significantly heightens the risk of undesirable color changes and surface wear in contrast to conventional whitening toothpastes (CWTs). Conversely, Alofi et al. [7], in an in vitro study involving 60 disc-shaped composite specimens, found no statistically significant difference in SR and color changes among CR discs after exposure to both CbDs and CWTs. Mehrgan et al. [5], in another in vitro study, conducted a comprehensive comparison of the effects of CbDs, hydrogen peroxide-containing dentifrices, and CWTs on the CS of CR exposed to coffee stains. The one-month follow-up revealed that, apart from CWTs, none of the dentifrices succeeded in reducing CR discoloration caused by the extrinsic coffee solution to a level below the perceptibility threshold [5]. Notably, CWTs effectively minimized CR discoloration within a clinically acceptable range [5]. A vigilant review of indexed literature showed that there is currently no documented systematic review of studies assessing the impact of CbDs on the CS and/or SR of CR.

With this background, the aim of the present systematic review was to assess studies that evaluated the effect of CbDs and CWTs on the CS and/or SR of CR. The question we focused on was “Do CbD and CWT affect the CS and/or SR of CR?”

## 2. Materials and Methods

### 2.1. Protocol and Focused Question

The present systematic review adhered to the guidelines outlined in the Preferred Reporting Items for Systematic Reviews and Meta-Analyses (PRISMA) [20]. To shape the specific focused question, the Population, Intervention, Control, and Outcome (PICO) framework was employed with the following components: P (Population) representing CR, I (Intervention) representing CbD, C (Control) representing CWT, and O (Outcome) encompassing the evaluation of CS and/or SR. The present study is registered with the Open Science Framework with the following registration DOI: https://doi.org/10.17605/OSF.IO/FUZCD.

### 2.2. Eligibility Criteria

Experimental peer-reviewed studies that assessed the impact of CbDs on the CS and/or SR of CR blocks were included. Case reports and case series, letters to the Editor, commentaries, pre-prints, reviews, retrospective studies, expert opinions, and perspectives were excluded.

### 2.3. Information Sources, Search Strategy, and Study Selection

An electronic search was conducted of indexed databases (PubMed [National Library of Medicine], EMBASE, Scopus, ISI Web of Knowledge, and Cochrane Library) without language and time restrictions from inception up to and including May 2023. The following medical subject terms were used: (1) charcoal-based; (2) activated charcoal; (3) charcoal; (4) toothpaste; (5) dentifrices; (6) bleaching; (7) oral hygiene; (8) enamel; (9) teeth; (10) composite resin; (11) CAD/CAM; (12) color stability; and (13) surface roughness. These keywords were combined using Boolean operators (OR/AND). Two authors (C.W. and F.J.) screened the titles and abstracts of the studies identified with the abovementioned protocol and independently read the full texts of relevant studies. The reference lists of pertinent original studies and review articles were searched to identify studies that might have been missed in the previous step. Disagreements were resolved via discussion with a third author (D.M.). The search strategy via databases and the list of excluded studies are shown in Supplementary Tables S1 and S2, respectively.

### 2.4. Data Collection and Data Items

The following data were extracted from eligible studies: reference; study design; number of samples (*n*); tinction; types of CR restoration block and dentifrice used; methods of evaluation of CS and SR; sample-size estimation (SSE); primary outcomes measured; allocation concealment (AC); blinding of investigators; and conclusion of study.

### 2.5. Risk of Bias in Individual Studies

The Quality Assessment Tool for In Vitro Studies (QUIN tool) was used for the risk of bias (RoB) assessment [21]. The QUIN Tool comprises the following 12 criteria: (a) clearly stated objectives; (b) detailed explanation of SSE; (c) detailed explanation of sampling technique; (d) details of comparison group; (e) detailed explanation of methodology; (f) operator details; (g) randomization; (h) method of measurement of outcome; (i) outcome assessor details; (j) blinding; (k) statistical analysis; and (l) presentation of results. Briefly, criteria that were “adequately specified”, “inadequately specified”, or “not specified” were allotted a score of “2”, “1”, and “0 or not applicable”, respectively. The total score for each study was determined and expressed as a percentage. Studies with scores of >70%, 50–70%, and <50% were graded as having a low, medium, or high RoB [21].

## 3. Results

### 3.1. Study Selection

**Figure 1:**
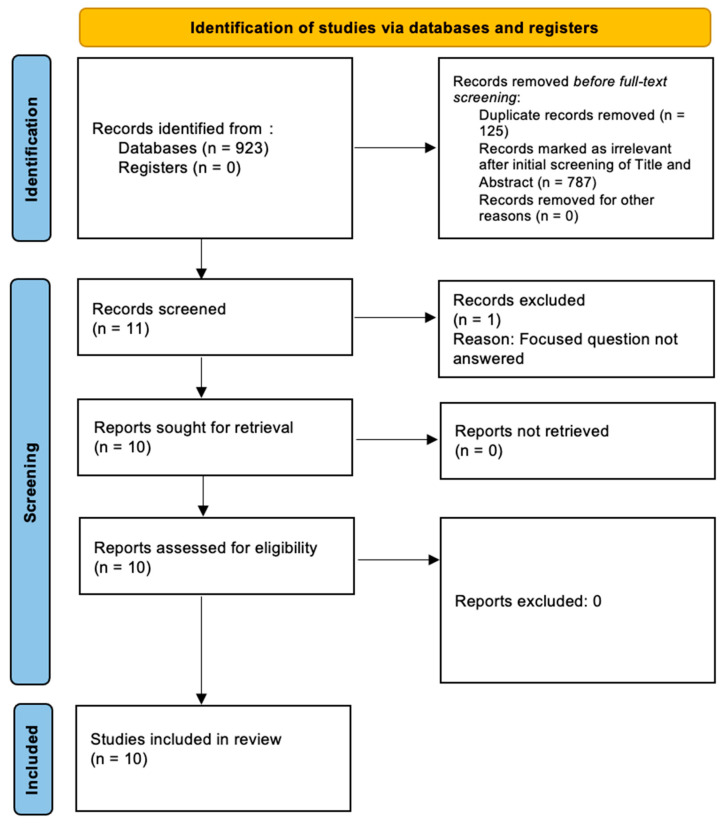
PRISMA flowchart.

The initial search yielded 923 studies. Of those, 125 duplicates were excluded. Of the remaining 798 studies, 54 were identified from EMBASE, 303 from PubMed, 169 from Scopus, 256 from Web of Science, and 16 from the Cochrane Library. Eleven full-text articles were screened, and one study that did not address the focused question was excluded. In total, ten in vitro studies [3,4,5,6,7,8,9,10,11,12] were included and processed for data extraction (Figure 1).

### 3.2. General Characteristics of the Included Studies

In five studies [6,7,8,9,10], the shade of CR used was A2. Pouryahya et al. [11], Aydin et al. [4], Binhasan et al. [12], and Mehrgan et al. [5] did not report the shade of composite used in their studies. The number of CR samples assessed for CS and/or SR ranged between 18 and 200. In six studies [4,5,6,7,8,9], CS was assessed using spectrophotometry, and in one study, a colorimeter was used [10]. Profilometers were used to assess SR in four studies [8,9,11,12]. In three studies [4,5,7], the tinction of CR was performed using a coffee solution; in another study [10], black tea was used for tinction. None of the remaining studies [3,8,9,12] reported the mode of composite tinction. Pouryahya et al. [11] and Rostamzadeh et al. [6] used accelerated artificial aging (AAA) to replicate composite aging. In one study [7], SR was assessed using a 3D optical microscope and non-contact surface metrology with interferometry, while in another study [10], an atomic force microscope was used (Table 1). Prior SSE was performed in one study [10]. In the study by Torso et al. [8], the length, width, and height of CR was 5 mm × 5 mm × 2 mm, whereas the diameter and depth of CR used by Bragança et al. [9] was 4 mm and 1 mm, respectively. Alofi et al. [7] used CR with a diameter and thickness of 100 and 2 mm, respectively, and Forouzanfar et al. [10] used CR with a diameter and thickness of 10 and 2 mm, respectively. Binhasan et al. [12] used CR with a height of 4 mm and a diameter of 10 mm. Mehrgan et al. [5], Pouryahya et al. [11], and Rostamzadeh et al. [6] all used CR with a diameter of 2 mm and a height of 7 mm. Aydin et al. [4] used CR with dimensions 1.5 mm × 7 mm × 12 mm. Law et al. [3] used CR with a diameter of 10 mm and a height of 1 mm. Alofi et al. [7] used Active Carbon, Yumaki^®^, for their CbD and Colgate^®^ Optic White^®^ Expert for their CWT. Torso et al. [8] used Colgate^®^ Total 12 as their CWT. In this study [8], four CbDs were used, namely Black is the new White; Natural Suavetex; Carvvo, L’aromatic; and Whitemax, Dermavita. Bragança et al. [9] used Bianco Pro Clinical as CWT and four CbDs, namely Bianco Carbon; Natural Suavetex; Nano Action Black Be Emotion; and Black is White. Forouzanfar et al. [10] used Colgate^®^ Max Fresh Cooling Crystals for their CWT and Colgate^®^ Max White Charcoal as their CbD. Pouryahya et al. [11] used Bencer, Perfect White Black, Colgate Optic White, and Colgate Total Whitening. Aydin et al. [4] used Oral B 3D White Luxe (Perfection), Colgate Optic White (Expert White), Signal White Now CC, Colgate Optic White (Charcoal), Splat Blackwood, and Sensodyne Deep Clean. Rostamzadeh et al. [6] used Colgate Optic White, Colgate Total Whitening, Perfect White Black, and Bencer charcoal. Mehrgan et al. [5] used Colgate Optic White, Colgate total whitening, Perfect white black, and Bencer charcoal. Binhasan et al. [12] used Colgate Optic White and Colgate Optic White Charcoal. Law et al. [3] used Crest 3D, Colgate Optic White, Hello (charcoal), and Aim.

### 3.3. Simulated Brushing Protocol

In four [8,9,10,12] of the included studies, toothbrushes with soft bristles were used. Mehrgan et al. [5] used a medium bristle toothbrush. Alofi et al. [7], Aydin et al. [5], Pouryahya et al. [11], and Rostamzadeh et al. [6] did not report the texture of bristles. In two studies [8,9], the powder/water ratio for CbDs was in the range of 1–8 g/3–4 mL of water. Two studies [7,10] did not precisely describe the powder/water dilution for CbDs. Binhasan et al. [12] reported a slurry of 50 g of toothpaste to 80 mL of deionized water used with a 180 g force at 2.5 cm/s speed. Pouryahya et al. [11] and Rostamzadeh et al. [6] both reported a slurry containing distilled water and dentifrice in a 50:50 ratio by weight. Rostamzadeh et al. [6] reported a brushing speed of 60 rpm. Mehrgan et al. [5] reported a toothpaste slurry of toothpaste and distilled water in a 3:1 ratio by weight. Aydin et al. [4] reported a slurry of distilled water and dentifrice in a 1:1 ratio by weight. For CWT, the powder/water ratio was in the range of 0.25–8 g/2–75 mL in three studies [8,9,10]. In the study by Alofi et al. [7], 0.25 mg of CWT was used as a paste slurry. In four studies [7,8,9,10], the brushing force ranged between 1.6 and 45 N. Pouryahya et al. [11] and Mehrgan et al. [5] did not report the brushing forces used, but back-and-forth motion within a 5 mm range was reported in both of these studies. Aydin et al. [4] did not report any brushing speed but did report a 2 N standardized force while brushing. In studies by Alofi et al. [7] and Forouzanfar et al. [10], brushing speeds and travel length (TL) were 35 mm/s and 99 mm/s and 15 mm and 4 mm, respectively. Law et al. [3] reported brushing at 150 gf for 10,000 cycles. Four studies [5,8,9,11] did not report brushing speed or TL (Table 2).

### 3.4. Outcomes

In the study by Alofi et al. [7], there were no statistically significant differences in the changes in the SR and CS of CR when CbDs were compared with CWTs, but CbDs did significantly increase the SR of CR (Table 3). Results by Torso et al. [8] showed that CbDs compromised the CS and SR of CR. According to Bragança et al. [9] CbDs significantly increased the SR of both CR groups; additionally, CbDs generally caused changes in the CS of CR when compared to CWTs. The results by Forouzanfar et al. [10] showed that CbDs increased SR and significantly reduced the CS of CR. The results by Pouryahya et al. [11] showed that there were no significant differences in surface roughness between CbDs and CWTs for CR samples. Aydin et al. [4] reported that there was no statistically significant difference between CbDs and CWTs when evaluating the CS of CR. Rostamzadeh et al. [6] showed that color parameter changes were not significantly different among the groups tested, but ΔE reached <3.3 only with CbD use and therefore reduced the CS of CR. Mehrgan et al. [5] reported that no statistically significant differences were found between the CS of CR when comparing CbDs and CWTs. Law et al. [3] reported that both CR groups tested were susceptible to the abrasive nature of both CbDs and CWTs. Finally, Binhasan et al. [12] reported that a significant increase in Ra was observed in CR after exposure to both CbDs and CWTs (Table 3).

### 3.5. Risk of Bias Assessment

Studies by Pouryahya et al. [11], Aydin et al. [4], and Law et al. [3] all had high RoB. The studies by Alofi et al. [7], Torso et al. [8], Binhasan et al. [12], Mehrgan et al. [5], and Forouzanfar et al. [10] had a medium RoB; and the studies by Bragança et al. [9] and Rostamzadeh et al. [6] had a low RoB (Table 4).

## 4. Discussion

In summary, the outcomes of the studies [3,4,5,6,7,8,9,10,11,12] included in the present review revealed varying outcomes. For instance, one study [7] demonstrated that there was no statistically significant influence on the SR and CS of CR regardless of whether CbDs were used or CWTs. In contrast, another study [8] showed that, unlike CWTs, the use of CbDs had adverse effects on CR, leading to an increase in SR and a compromise in CS. The findings from Bragança and colleagues [9] indicated that CbDs could alter CS when compared to CWTs; however, there was no discernible difference in the SR of CR induced by both dentifrices. Introducing additional intricacy to the issue, the study by Forouzanfar et al. [10], which examined the impact of CbDs and CWTs on the SR of CR reported that CWTs as well as CbDs increased the SR of CR. However, it is crucial to note that a comprehensive statistical analysis could not be conducted on this dataset due to varying protocols and inconsistent materials and methods. The authors of the current study encountered confusion regarding the statement made by Forouzanfar et al. [10] as this was the only study that was conducted with prior SSE. Consequently, the statement in the study by Forouzanfar et al. [10], which read “due to lack of specimens’ number, the statistical analysis could not be done on these data,” appears perplexing and challenging to comprehend in light of their meticulous SSE approach. Nevertheless, the extraction of substantial comparative outcomes, particularly in cases where quantitative parameters pertaining to SR are not subjected to rigorous statistical assessment, becomes a challenging endeavor. It is also worth mentioning that 60% of the studies [4,5,6,7,8,9] included in the present review used visual spectrophotometry (VS) for the assessment of change in CS. Kayahan et al. [22] reported that vs. is considered a “gold standard technique for assessment of CS due to its ability to detect even subtle alterations in color, its ability to produce consistent results, and its objectivity.” While a colorimeter (used for the detection of CS in the study by Forouzanfar et al. [10]) can provide valuable information and is more affordable and user-friendly, vs. is generally considered more accurate and versatile due to its ability to measure a broader range of color changes [23,24]. These factors appear to have contributed to an elevated risk of bias in the study conducted by Forouzanfar et al. [10]. While the latter study underwent power adjustment, the authors of the present review recommend exercising caution when interpreting the reported outcomes. Moreover, the diverse outcomes reported in the included studies [7,8,9,10] suggest a need for further investigation and consideration of the specific characteristics of dentifrices in relation to their impact on the CS and SR properties of CR.

The translation of the experimental findings [3,4,5,6,7,8,9,10,11,12] for potential application in clinical contexts presents a noteworthy challenge primarily due to the potential influence of various factors on the reported outcomes. It is essential to emphasize that the experimental studies [3,4,5,6,7,8,9,10,11,12] encompassed within the scope of the present systematic review utilized inconsistent shades of CR. Within the domain of operative and restorative dentistry, CR materials are commercially available in a spectrum of distinct shades, each characterized by varying degrees of translucency. Akbar et al. [25] provided empirical evidence indicating a substantial decline in translucency when transitioning from A2 to D2 shades, as well as a decrease in diffuse translucency when progressing from A4 to C6 shades of CR. The authors concluded that the coloration of CR exerts a discernible and statistically significant impact on its translucency characteristics [25]. The authors of the present investigation hypothesize that the changes in the CS of CR after brushing with CbDs or CWTs vary depending upon the original shade and translucency of CR. Further studies are needed to test this hypothesis.

A noteworthy observation within the included studies [3,4,5,6,7,8,9,10,11,12] is the inconsistent utilization of similar firmness toothbrushes for the brushing of CR. In a clinical context, it is imperative to acknowledge that individuals may employ toothbrushes featuring a spectrum of bristle firmness levels, encompassing soft, medium, or hard, which reduces the chances of coming to a sound conclusion based on the existing evidence from the included studies [3,4,5,6,7,8,9,10,11,12] due to the wide variation in abrasiveness found in the toothbrushes used. Some studies [14,15,26] have reported that the duration of brushing represents a more significant determinant impacting SR of CR than toothbrush bristle hardness. It is also pertinent to note that the microstructure and composition of CR represent additional factors that exert influence upon SR [27]. According to Ferracane et al. [28], the optical and physical characteristics of CR are significantly governed by key factors such as the nature, dimensions, quantity, and dispersion of resin-reinforcing fillers. It is crucial to emphasize that, within the studies [3,4,5,6,7,8,9,10,11,12], there was an absence of standardized specifications pertaining to the dimensions of CR. Moreover, the precise composition and filler content of the assessed CR samples were not reported. Furthermore, within clinical contexts, individuals characterized by persistent exposure to extrinsic staining agents, as observed in habitual nicotine-containing product users and those with excessive intake of staining beverages such as coffee and tea, may introduce complexities in the endeavor to ascertain the impacts of dentifrices such as CbDs and CWTs on the CS of CR. In this regard, further studies are needed to assess the effect of additional variables such as those referenced above on the CS and SR of CR following brushing with CbDs and CWTs.

A limitation of the present systematic review is that a quantitative assessment (meta-analysis) of data from eligible studies [3,4,5,6,7,8,9,10,11,12] was not executed. The primary reasoning for this is the methodological heterogeneity encompassing aspects such as the dimensions of CR, the quantity of dentifrice applied, the number of brushing cycles, and the firmness of the toothbrushes used. Moreover, the protocol of the present SR could not be registered in the International Prospective Register of Systematic Reviews (PROSPERO). It is worth mentioning that PROSPERO permits the registration of systematic reviews on either human subjects or animal models [29]. It is also worth mentioning that none of the studies that fulfilled the eligibility criteria assessed the relative dentin abrasivity (RDA) of the dentifrices. The RDA is a measure used to assess the abrasive potential of a dentifrice, which indicates how abrasive a toothpaste is to dentin [30]. The RDA is assigned a numerical value, and the higher the value, the more abrasive the dentifrice [30]. Nevertheless, since the present systematic review focused on the CS and SR of composites, an assessment of the RDA was deemed outside the scope of the present investigation. Furthermore, Cohen’s Kappa score for inter-rater reliability was not calculated in the present systematic review. This decision was based on the nature of the present systematic review and the specific characteristics of the included studies [3,4,5,6,7,8,9,10,11,12]. The inherent complexity and diversity in study designs, methodologies, and outcome measures made the application of Cohen’s kappa less suitable for a comprehensive assessment of inter-rater reliability in our specific context. Furthermore, the presence of a high/medium RoB in 80% of the studies [3,4,5,7,8,10,11,12] and a deficiency in prior SSE in 90% [3,4,5,6,7,8,9,11,12] of the studies further complicated the task of achieving a reliable interpretation of the reported outcomes.

## 5. Conclusions

Compared to CWTs, CbDs appeared to affect the CS of CR but the SR of CR induced by both dentifrices remained consistent. Further well-designed and power-adjusted studies are needed to further comprehend the effect of CbDs and CWTs in the CS and SR of CR.

## Figures and Tables

**Table 1:** General characteristics of included in vitro studies.

Study	Tinction	Composite Shade	Samples Assessed for CS (*n*)	Samples Assessed for SR (*n*)	Test-Group	Control-Group	Mode of CS Evaluation	Mode of SR Evaluation
Alofi et al. [7]	Coffee solution	A2	33	33	CbD	CWT	Spectrophotometer	A 3D optical microscope and non-contact surface metrology with interferometry
Torso et al. [8]	NA	A2	35	25	CbD	CWT	Spectrophotometer	Profilometric analysis
Bragança et al. [9]	NA	A2	100	100	CbD	CWT	Spectrophotometer	Profilometric analysis
Forouzanfar et al. [10]	Black Tea	A2	18	18	CbD	CWT	Colorimeter	Atomic force microscope
Rostamzadeh et al. [6]	AAA	A2	45	NA	CbD	CWT	Spectrophotometer	NA
Pouryahya et al. [11]	AAA	NA	NA	45	CbD	CWT	NA	Profilometer
Binhasan et al. [12]	NA	NA	NA	96	CbD	CWT	NA	Profilometer
Aydin et al. [4]	Coffee solution	NA	200	NA	CbD	CWT	Spectrophotometer	NA
Mehrgan et al. [5]	Coffee solution	NA	45	NA	CbD	CWT	Spectrophotometer	NA
Law et al. [3]	NA	NA	NA	64	CbD	CWT	NA	Profilometer

CbD: charcoal-based dentifrice; CWT: commercial whitening toothpaste; CS: color stability; NA: not available; SR: surface roughness; AAA = accelerate artificial aging.

**Table 2:** Protocol of simulated brushing.

Study	Bristle Texture	Amount of CbD	Amount of CWT	Brushing Cycles	Brushing Force	Brushing Speed	Travel Length
Law et al. [3]	NR	37.5 g/60 g water	37.5 g/60 g water	10,000 cycles	150 g	NR	NR
Aydin et al. [4]	NR	1:1 ratio	1:1 ratio	NR	2 N	NR	NR
Mehrgan et al. [5]	Medium	3:1 ratio	3:1 ratio	120 cycles	NR	NR	5 mm
Rostamzadeh et al. [6]	NR	50:50 ratio	50:50 ratio	NR	NR	60 rpm	5 mm
Alofi et al. [7]	NR	0.25 mg in water (1:3 ratio)	0.25 mg paste slurry	1120 cycles	1.6 N	35 mm/s	15 mm
Torso et al. [8]	Soft	4 mL in deionized water (1:3 ratio)	0.25 g dissolved in 75 mL water	417 and 5004 cycles	1.96 N	NR	NR
Bragança et al. [9]	Soft	8 g/4 mL artificial saliva (2:1 ratio)	8 g/4 mL artificial saliva (2:1 ratio)	21,960 cycles	200 g	NR	NR
Forouzanfar et al. [10]	Soft	1 g/3 mL water (1:3 ratio)	1 g/3 mL water (1:3 ratio)	10,000 cycles	45 N	99 mm/s	4 mm
Pouryahya et al. [11]	NR	50:50 ratio	50:50 ratio	240 cycles/min	NR	NR	5 mm
Binhasan et al. [12]	Soft	50 g/80 mL	50 g/80 mL	25,000 strokes	180 g	2.5 cm/s	NR

CbD: charcoal-based dentifrice; CWT: commercial whitening toothpaste; NR: not reported; mm/s: millimeters/second; mm: millimeters.

**Table 3:** Study outcomes for CbD use on CR.

Study	Samples	Composite Resin	Effects on SR	Effects on CS
Law et al. [3]	64 SR	Spectra TPH (*n* = 32) Filtek Bulk Fill (*n* = 32)	CbD significantly increased the SR of CR.	NA
Aydin et al. [4]	200 CS	Resin-based CAD/CAM	NA	CbD did not significantly reduce the CS of CR.
Mehrgan et al. [5]	45 CS	Spectra TPH	NA	CbD did not significantly reduce the CS of CR.
Rostamzadeh et al. [6]	45 CS	Spectra TPH3	NA	CbD significantly reduced the CS of CR.
Alofi et al. [7]	33 CS; 33 SR	IPS empress direct composite	CbD significantly increased the SR of CR.	CbD significantly reduced the CS of CR.
Torso et al. [8]	35 CS; 25 SR	Filtek Z350	CbD significantly increased the SR of CR.	CbD significantly reduced the CS of CR.
Bragança et al. [9]	100 CS; 100 SR	Filtek Z350 XT (*n* = 100) Vittra APS (*n* = 100)	CbD significantly increased the SR of both CR groups.	CbD significantly reduced the CS of CR.
Forouzanfar et al. [10]	18 CS; 18 SR	Filtek Z250	CbD increased the SR of CR.	CbD significantly reduced the CS of CR.
Pouryahya et al. [11]	45 SR	Spectr TPH3	CbD did not significantly increase the SR of CR.	NA
Binhasan et al. [12]	96 SR	Filtek Z350 XT (*n* = 48) Tetric N Ceram Bulk-fill (*n* = 48)	CbD significantly increased the SR of both CR groups.	NA

CS: color stability; SR: surface roughness; CbD: charcoal-based dentifrice.

**Table 4:** Risk of bias assessment using the Quality Assessment Tool for In Vitro Studies (QUIN tool).

Criteria	Law et al. [3]	Aydin et al. [4]	Mehrgan et al. [5]	Rostamzadeh et al. [6]	Alofi et al. [7]	Torso et al. [8]	Bragança et al. [9]	Forouzanfar et al. [10]	Pouryahya et al. [11]	Binhasan et al. [12]
Clearly stated objectives	1	2	2	2	2	2	2	2	2	2
Detailed explanation of sample-size calculation	0	0	0	1	0	0	0	2	0	0
Detailed explanation of the sampling technique	2	1	2	2	2	2	2	2	1	2
Details of the comparison group	2	2	2	2	2	2	2	2	1	2
Detailed explanation of the methodology	1	1	2	2	1	1	1	2	1	2
Operator details	0	0	0	0	0	0	0	0	0	0
Randomization	0	0	0	0	1	1	1	0	0	0
Method of measurement of outcome	2	2	2	2	2	2	2	1	2	2
Outcome assessor details	2	2	2	2	2	2	2	2	2	2
Blinding	0	0	0	0	0	0	1	0	0	0
Statistical analysis	1	1	1	2	2	2	2	1	1	2
Presentation of results	2	1	2	2	2	1	2	1	2	2
Total score	13	12	15	17	16	15	17	16	12	16
Percentage	54.1%	50%	62.5%	70.8%	66.6%	62.5%	70.8%	62.5%	50%	62.5%

## Data Availability

Data are available upon reasonable request.

## Supplementary Materials

The following supporting information can be downloaded at: https://www.mdpi.com/article/10.3390/dj12030058/s1, Table S1: Search databases and search strategies; Table S2: Excluded studies. Reference [31] is cited in the Supplementary Materials.
