# Supplementary material for: The Effect of Charcoal-Based Dentifrice and Conventional Whitening Toothpaste on the Color Stability and Surface Roughness of Composite Resin: A Systematic Review of In Vitro Studies
Source: Dent J (Basel). 2024 Mar 1;12(3):58. doi: 10.3390/dj12030058 (PMC10969454; doi:10.3390/dj12030058)
Supplement: Supplementary file 1 [file dentistry-12-00058-s001.zip › dentistry-2711221-supplementary.pdf]

**Supplementary Table S1.** Search databases and search strategies

| Search Database | Date Searched | Search Terms | Number of Results |
| --- | --- | --- | --- |
| PubMed | 09/20/23 | (charcoal-based OR activated charcoal OR charcoal OR soot) AND (toothpaste OR dentifrices OR bleaching OR oral hygiene OR enamel OR teeth OR resin composites OR CAD/CAM OR bleaching OR oral hygiene OR color stability OR surface roughness) | 303 |
| EMBASE | 12/29/23 | ('charcoal based' OR 'activated charcoal' OR charcoal OR soot) AND ((toothpaste OR dentifrices OR bleaching OR oral) AND hygiene OR enamel OR teeth OR 'resin composites' OR 'CAD CAM' OR bleaching OR 'oral hygiene' OR 'color stability' OR 'surface roughness') | 54 |
| Scopus | 12/29/23 | ( {charcoal based} OR {activated charcoal} OR charcoal OR soot ) AND ( toothpaste OR dentifrices OR bleaching OR {oral hygiene} OR enamel OR teeth OR {resin composites} OR {CAD CAM} OR bleaching OR {oral hygiene} OR {color stability} OR {surface roughness} ) AND ( LIMIT-TO ( SUBJAREA , "DENT" ) ) | 169 |
| ISI Web of Knowledge | 12/29/23 | (ALL=((toothpaste OR dentifrices OR bleaching OR "oral hygiene" OR enamel OR teeth OR "resin composites" OR "CAD CAM" OR bleaching OR "oral hygiene" OR "color stability" OR "surface roughness"))) AND ALL=(("charcoal based" OR "activated charcoal" OR charcoal OR soot)) | 256 |
| Cochrane Library | 12/29/23 | (charcoal-based OR activated charcoal OR charcoal OR soot) AND (toothpaste OR dentifrices OR bleaching OR oral hygiene OR enamel OR teeth OR resin composites OR CAD CAM OR bleaching OR oral hygiene OR color stability OR surface roughness) | 16 |

**Supplementary Table S2.** Excluded studies.

| **Study** | Reason for Exclusion |
|---|---|
| Pouryahya R, Ranjbar Omrani L, Ahmadi E, Rafeie N, Abbasi M. Effect of charcoal-based dentifrices on surface roughness of an aged resin composite. Minerva Dent Oral Sci. 2023 Feb;72(1):24-30. doi: 10.23736/S2724-6329.21.04613-1. | Did not evaluate color stability |
| Aydın N, Karaoglanoglu S, Oktay EA. Investigation the effects of whitening toothpastes on color change of resin-based CAD/CAM blocks. J Esthet Restor Dent. 2021 Sep;33(6):884-890. doi: 10.1111/jerd.12627. Epub 2020 Jul 23. | Did not evaluate surface roughness |
| Mehrgan S, Kermanshah H, Omrani LR, Ahmadi E, Rafeie N. Comparison the effect of charcoal-containing, hydrogen peroxide-containing, and abrasive whitening toothpastes on color stability of a resin composite; an in vitro study. BMC Oral Health. 2021 Nov 19;21(1):594. doi: 10.1186/s12903-021-01956-8. | Did not evaluate surface roughness |
| Köroğlu A, Şahin O, Küçükekenci AS, Dede DÖ, Yıldırım H, Yilmaz B. Influences of Toothbrushing and Different Toothpastes on the Surface Roughness and Color Stability of Interim Prosthodontic Materials. Materials (Basel). 2022 Aug 24;15(17):5831. doi: 10.3390/ma15175831. | Interim prosthodontic crown materials used |
| Rostamzadeh P, Omrani LR, Abbasi M, Yekaninejad MS, Ahmadi E. Effect of whitening toothpastes containing activated charcoal, abrasive particles, or hydrogen peroxide on the color of aged microhybrid composite. Dent Res J (Isfahan). 2021 Dec 10;18:106. | Did not evaluate surface roughness |
| Law V, Levy LC, Morrow BR, Garcia-Godoy F. Effect of whitening dentifrices on toothbrush abrasion on composites. Am J Dent. 2022 Jun;35(2):161-164. PID: | Did not evaluate color stability |
| Binhasan M, Solimanie AH, Almuammar KS, Alnajres AR, Alhamdan MM, Al Ahdal K, Alfaawaz YF, Ali K, Vohra F, Abduljabbar T. The Effect of Dentifrice on Micro-Hardness, Surface Gloss, and Micro-Roughness of Nano Filled Conventional and Bulk-Fill Polymer Composite-A Micro Indentation and Profilometric Study. Materials (Basel). 2022 Jun 20;15(12):4347. doi: 10.3390/ma15124347. | Did not evaluate color stability |
